# Binding Site Graphs: A New Graph Theoretical Framework for Prediction of Transcription Factor Binding Sites

**DOI:** 10.1371/journal.pcbi.0030090

**Published:** 2007-05-11

**Authors:** Timothy E Reddy, Charles DeLisi, Boris E Shakhnovich

**Affiliations:** 1 Program in Bioinformatics and Systems Biology, Boston University, Boston, Massachusetts, United States of America; 2 Department of Biomedical Engineering, Boston University, Boston, Massachusetts, United States of America; 3 Department of Molecular and Cellular Biology, Harvard University, Cambridge, Massachusetts, United States of America; Washington University of St. Louis, United States of America

## Abstract

Computational prediction of nucleotide binding specificity for transcription factors remains a fundamental and largely unsolved problem. Determination of binding positions is a prerequisite for research in gene regulation, a major mechanism controlling phenotypic diversity. Furthermore, an accurate determination of binding specificities from high-throughput data sources is necessary to realize the full potential of systems biology. Unfortunately, recently performed independent evaluation showed that more than half the predictions from most widely used algorithms are false. We introduce a graph-theoretical framework to describe local sequence similarity as the pair-wise distances between nucleotides in promoter sequences, and hypothesize that densely connected subgraphs are indicative of transcription factor binding sites. Using a well-established sampling algorithm coupled with simple clustering and scoring schemes, we identify sets of closely related nucleotides and test those for known TF binding activity. Using an independent benchmark, we find our algorithm predicts yeast binding motifs considerably better than currently available techniques and without manual curation. Importantly, we reduce the number of false positive predictions in yeast to less than 30%. We also develop a framework to evaluate the statistical significance of our motif predictions. We show that our approach is robust to the choice of input promoters, and thus can be used in the context of predicting binding positions from noisy experimental data. We apply our method to identify binding sites using data from genome scale ChIP–chip experiments. Results from these experiments are publicly available at http://cagt10.bu.edu/BSG. The graphical framework developed here may be useful when combining predictions from numerous computational and experimental measures. Finally, we discuss how our algorithm can be used to improve the sensitivity of computational predictions of transcription factor binding specificities.

## Author Summary

A historically difficult problem in computational biology is the identification of transcription factor binding sites (TFBS) in the promoters of co-regulated genes. With increasing emphasis on research in transcriptional regulation, this problem is also uniquely relevant to emerging results from recent experiments in high-throughput and systems biology. Despite extensive research in the area, recent evaluations of previously published techniques show much room for improvement. In this paper, we introduce a fundamentally new approach to the identification of TFBS. First, we start by representing nucleotides in promoters as an undirected, weighted graph. Given this representation of a binding site graph (BSG), we employ relatively simple graph clustering techniques to identify functional TFBS. We show that BSG predictions significantly outperform all previously evaluated methods in nearly every performance measure using a standardized assessment benchmark. We also find that this approach is more robust than traditional Gibbs sampling to selection of input promoters, and thus more likely to perform well under noisy experimental conditions. Finally, BSGs are very good at predicting specificity determining nucleotides. Using BSG predictions, we were able to confirm recent experimental results on binding specificity of E-box TFs CBF1 and PHO4 and predict novel specificity determining nucleotides for TYE7.

## Introduction

Transcription factors (TFs) bind short stretches (usually 6–18 bp) of DNA near the gene's transcription start site. This event is thought to facilitate regulation of expression of the downstream gene through TF interaction with the RNA polymerase and other factors in the pre-initiation complex [1]. Computational identification of transcription factor binding sites (TFBS) remains one of the most challenging and important problems at the interface of computational and experimental research. In general, research in a diverse array of fields from biophysics to systems biology often depends on the ability to accurately identify TF binding propensities and positions. For example, several models of promoter architecture require knowledge of binding locations to identify transcriptional logic gates [2] defined, in part by the relative binding positions of TFs [3,4].

The main in vivo approaches to TF binding site determination are variants of ChIP–chip assays, and DNA footprinting. The former, which is essentially a high-throughput version of the latter, can identify approximate location of binding, usually accurate enough to within the length of a promoter [5,6]. Footprinting can provide exact binding positions, but it is not easily generalized to high-throughput studies [7]. Thus, to identify binding positions at the genomic scale, researchers often combine high-throughput ChIP–chip experiments with computational algorithms to predict TF binding sites and nucleotide affinities. Developing TF:DNA binding models from first principles, however, is complicated by limited understanding of mechanisms governing transcription factor binding and subsequent transduction of the polymerase assembly. Instead, several empirically derived models have been proposed to identify biologically relevant stretches of promoter regions [8–12]. Most computational algorithms depend on experimental assays to identify sets of co-regulated genes and work by recognizing over-represented, short stretches of DNA.

A recent evaluation of some of these algorithms shows that computational treatment of TFBSs is a largely unsolved problem, with the majority of tested algorithms predicting less than 50% of binding sites correctly [13]. Several well-known pitfalls intrinsic to both the biological and computational sides of this problem plague algorithmic identification of binding positions. First, the possible space of solutions is very large, while heuristic approaches often identify local optima [14]. Even if the algorithms could reliably identify global optima, empirically derived scoring functions do not reliably select biologically significant binding sites. Furthermore, the number of bound sites is close to that which could occur by random chance given the length of most eukaryotic promoters [15], making identification by statistical over-representation challenging. The variability in DNA sequence that retains TF function and allows regulation of the expression of the downstream gene is unknown. While distance relative to the transcription start site was recently shown to be important [16], this observation is not specific enough to apply in an algorithmic sense to TFBS identification. Finally, while the range of widths that TFs bind is largely accepted to be between 6 and 18 bp, an unbiased estimation for the width of the sequence specific to individual TFs has proven especially difficult [17,18].

Recent innovations in computational TF motif prediction have attempted to incorporate orthogonal information to improve predictions. Position-specific mutation models [19,20], co-occurrence of binding sites for multiple TFs [21], and phylogenetic conservation [20,22,23], among other approaches [11,24], have been proposed as additional measures. While all these measures can be shown to improve either accuracy or coverage of computational predictions, most introduce biases that may narrow their applicability. For example, requiring strict phylogenetic conservation automatically excludes identification of evolutionary changes of transcriptional regulation [16], and those that rely on co-occurrence of different sites do not help with identifying binding of a single TF of interest. Several researchers have also outlined a strategy utilizing the consensus from a variety of programs to improve accuracy [5]. However, the improvement in accuracy of predictions from adopting this approach has not been rigorously quantified and consequently not well understood.

Here, we build on existing computational approaches to improve prediction of TF binding positions without adding additional biases that may narrow the scope of application. We recently showed [25] that extensive repetition of Gibbs sampling on the same set of upstream promoters, termed ensemble Gibbs sampling (see Methods), yields a power-law distribution of hits per nucleotide in each promoter: few nucleotides are selected very frequently, while the majority of nucleotides (also representing the majority of the Gibbs sampling results) are identified infrequently and do not correspond to biologically relevant results. Simple positional clustering to select the most frequently recurring nucleotides improves accuracy of TFBS identification [25]. Here we show positional clustering can be substantially improved by considering joint occurrences of nucleotides in the same motif. These joint probabilities can be represented as a binding site graph (BSG).

Using a well-established benchmark, we compare the predictive power of BSGs to 13 other TFBS prediction algorithms [13]. On yeast datasets, BSGs significantly outperform all previously evaluated algorithms in nearly every measure. In particular, the high percentage of correct predictions (PPV, positive predictive value) indicates that the approach is useful for directing downstream experimental research. Performance on non-yeast benchmarks, however, is dramatically worse, signifying that more research is required to reliably predict fly and mammalian regulatory motifs. We also find that BSG predictions are robust to the choice and length of input promoters, and thus more likely to succeed with limited or noisy experimental data. Encouraged by performance on yeast benchmarks, we use BSGs to predict the condition-specific nucleotide specificity for most known TFs in the Saccharomyces cerevisiae genome. Predictions for previously characterized TFs closely agree with previous experimental and computational studies. In addition, we predict 53 novel binding specificities, 16 at high statistical significance.

## Results

**Figure 1:**
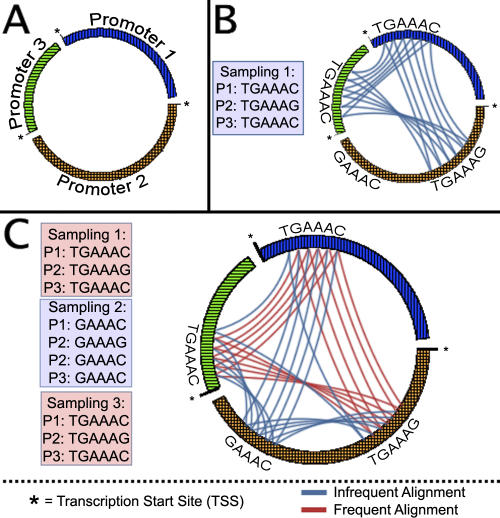
BSG Construction (A) Vertices of the BSG represent nucleotides in input promoters. Here, the input nucleotides are represented as points along the perimeter of a circle. Each disconnected bar along the perimeter of the circle represents a single promoter, with the transcription start site indicated. (B) Edges (arcs across the circle) are added to each pair of aligned nucleotides in a motif resulting from a single Gibbs sampling prediction. (C) Edges are compiled across ensemble Gibbs sampling results. Recurring edges are weighted by the number of times they recur, as indicated by different colored arcs in the BSG. Once all edges are collected from ensemble Gibbs sampling results, edge weights are normalized by the most frequently recurring edge.

**Figure 2:**
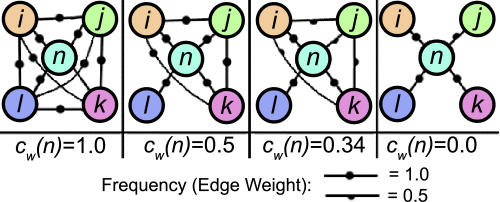
An Illustration of the Weighted Clustering Coefficient: Behavior of the Weighted Clustering Coefficient of Vertex *n* Is Shown across Four Increasingly Sparse Graphs (A) In the most dense case, each pair of vertices adjacent to *n* is also adjacent, and all edge weights are maximal. Thus, *n* participates in a maximally weighted clique, and the weighted clustering coefficient is 1. (B) As edges are removed from the clique, the neighborhood of *n* becomes less well-connected, and the clustering coefficient decreases. (C) Unlike the original definition of clustering coefficient, the weighted clustering coefficient responds to the decreased intensity of the cluster resulting from intermediately weighted edges. (D) Finally, when no edges exist between neighbors of *n,* the clustering coefficient goes to 0.

A BSG is a graphical model representing the pair-wise nucleotide co-occurrences in the same motif. Vertices in a BSG represent nucleotides in the input promoters, and edges are weighted by an estimated probability of nucleotide co-occurrence in the same TF binding motif. Edges can be estimated using a variety of techniques: here, we use ensemble Gibbs sampling [25] to construct a BSG (Figure 1). Doing so, we weight the edges by the fraction of the Gibbs sampling predictions in which two nucleotides co-occur (Figure 1C). Once a BSG is constructed, it remains to identify the subgraph of densely connected nucleotides corresponding to TF binding sites [26]. Various graph properties and clustering techniques may be useful to identify such clusters. We focus on the frequency with which pairs of nucleotides in the cluster co-occur (represented in edge weights), and the extent to which the cluster is interconnected (measured by the clustering coefficient, or cliquishness, of the cluster). However, sets of nucleotides that are always identified together in Gibbs sampling, but do so in very few Gibbs sampling results, are more likely the result of noise in Gibbs sampling results than of correct predictions [25]. Therefore, we employ a generalization of the clustering coefficient to weighted graphs that evaluates both the number and the weight of edges in a cluster of nucleotides [27]: thus, such a low-weight clique of nucleotides will receive a very small weighted clustering coefficient (Figure 2). To evaluate the quality of a prediction, we develop a binding site graph score (BSGscore) that takes into account both the weighted clustering coefficient and the size of the subgraph. Comparing to a background distribution of sets of randomly selected promoters, we go on to evaluate the statistical significance of a BSGscore.

### BSG Construction and Prediction of TFBS

Given an input set of promoters, we construct a BSG from ensemble Gibbs sampling, as shown in Figure 1. Briefly, for each dataset, we run the sampler until stability 512 times for each motif width 6–18 bp, producing a total of 6,656 predictions per dataset. We consider the sampler to reach stability when results do not change over 1,250 updates. For each prediction, we add an edge between all pairs of nucleotide positions in the same column of the returned multiple sequence alignment (Figure 1B). Edges recurring in multiple Gibbs sampling results are represented by a single edge with weight *w* equal to the number of times the edge occurs normalized by the total number of Gibbs sampling predictions. Thus, *w* ∈ (0,P], where the maximum edge weight, P ≤ 1, is the number of times the most frequently recurring edge is observed divided by the total number of edges in the graph.

Once the BSG is built, it still remains to predict positions corresponding to functional TFBS. First, analogous to the frequency with which Gibbs sampling identifies a given nucleotide [25], edge weights are power law distributed (unpublished data), and nucleotides connected with high edge weights are predictive of TFBS (Figure S1). Second, we hypothesize that transitively connected nucleotides are closely related in sequence space. For example, if Gibbs sampling identified sites 1 and 2 in one run and sites 2 and 3 in another, those sites will have related, but not identical, sequences. We are interested in differentiating the case where the Gibbs sampler identifies random sets of k-mers from the case where the sampler repeatedly predicts the same set of sites. We hypothesize that the latter case corresponds to functional TFBS. This information is represented in BSG by dense clusters of nucleotides connected by high edge weights.

The clustering coefficient of a nucleotide *k* in a BSG measures connectivity within the local neighborhood of *k* [28]. As the neighborhood of *k* approaches a clique, where all neighbors are connected, the clustering coefficient approaches 1. As the neighborhood of *k* becomes sparse, where no neighbors of *k* co-occur in Gibbs sampling predictions, the clustering coefficient approaches 0. The standard definition of the clustering coefficient is limited to unweighted graphs. However, because edge weights are predictive of functional TFBS, we use a modified version of the clustering coefficient that rewards higher edge weights [27] (Figure 2).

**Figure 3:**
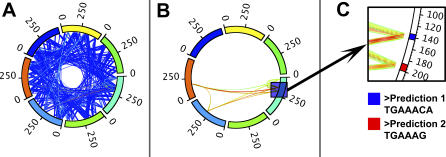
Predicting TFBS within the BSG Framework (A) First, a BSG is constructed from ensemble Gibbs Sampling. Here, the perimeter of the circle represents promoters, and lines between nucleotides in the promoters correspond to edges in the BSG. Edges are heat-mapped according to edge weight. (B) The filtered BSG, obtained by selecting an edge-weight threshold ρ to maximize the BSGscore, followed by all edges with weight less than ρ from the graph. (C) Final TFBS predictions are made from the filtered BSG by collecting nucleotides contiguous in the original promoters into prediction sequences, which are returned in fasta format. The promoter region depicted contains two predicted TFBS.

To predict TFBS from a BSG, we can use the weighted clustering coefficient to find sets of nucleotides that are densely connected with high-weight edges. We will use a threshold 0 < *ρ* ≤ P to filter out all edges with inconsequential edge weight (Figure 3). Since dense clusters are more likely to occur at random in graphs with fewer vertices, simply maximizing on the weighted clustering coefficient is biased toward graphs with the fewest nodes (Figure S3). To account for this, we include a (1 − *ρ/* P) term in our BSGscore to reward larger but perhaps less densely connected subgraphs. Thus, for a BSG *G* and frequency threshold *ρ,* we define the BSGscore:

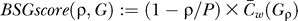
where 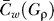 is mean weighted clustering coefficient over all nucleotides in graph *G* at threshold *ρ*. We want to select the edge weight threshold ρˆ that maximizes the BSGscore. To turn the resulting graph into predicted binding positions in the promoters, we extract all nucleotides in the BSG connected by an edge with *w* > ρˆ (Figure 3B). Nucleotides adjacent in the original input promoters are grouped together into seed sequences (see Methods, Figure 3C). Often, we find the seed sequences contain only the most conserved core of the TFBS. To capture important nucleotides at the edges, we extend the seed sequences to include neighboring nucleotides that are also frequently identified by Gibbs sampling, but perhaps do not pass the stringent cutoff of the core positions (see Methods). The extended sequences are then aligned to create a position weight matrix (PWM) representing the binding motif.

It should be noted that, at this point in the process, the remaining sequences are generally of similar length, and well-conserved. Hence, the primary motivation for using a sampling procedure here is not to define the end points of the alignment. Instead, we use sampling to solve the problem that we do not know the strand orientation of each binding site. The space of all possible permutations of strand orientations is exponential, and it is unfeasible to explore exhaustively for even a moderate number of predictions. Thus, we use sampling to heuristically predict the relative strand orientation of the predictions. Indeed, other procedures such as expectation maximization would also be appropriate here, and Gibbs sampling was chosen as a matter of convenience.

**Figure 4:**
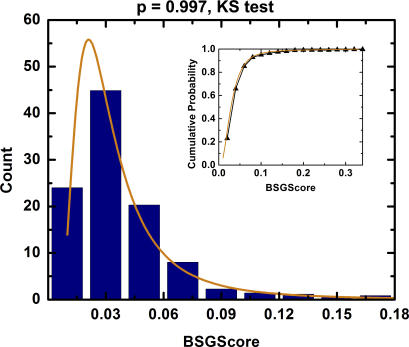
Frequency (*y*-Axis) of BSGscores (*x*-Axis) for Set of 7–30 Randomly Selected Yeast Promoters (Blue Bars) Compared with the Probability Distribution of the Estimated Generalized Extreme Value Distribution (Orange Line) Empirical and estimated cumulative probability distribution (black triangles and orange line, respectively) are shown in the inset. The empirical and estimated distributions are the same with *p* = 0.997 according to a KS test.

To evaluate the statistical significance of a BSG prediction, it is important to understand the behavior of BSGscores under the null hypothesis that no motifs are present in the input set. We estimated the null distribution of BSGscores in yeast by calculating the maximal BSGscore from 429 sets of 7–30 randomly chosen (without replacement) yeast promoters. The resulting scores follow a generalized extreme value distribution (Figure 4; *p* = 0.997, KS test). We found no significant correlation between the size of the random input set and the BSGscore. Using this distribution, we can evaluate the *p*-value of a BSGscore from a dataset enriched in a TF binding motif (see Methods). However, dramatic differences in promoter architecture between species may mean that an empirically derived background distribution is needed on a per-species basis. Additionally, we leave for future study a statistical evaluation of input sets with multiple motifs.

### Whole Genome TFBS Predictions from ChIP–chip in S. cerevisiae

We used BSGs to predict binding sites for the majority of TFs in the S. cerevisiae genome using the latest data from ChIP–chip experiments [5] in a number of experimental conditions. In total, BSGs predict significant binding motifs for 118 TF–condition pairs, representing 93 different TFs. These results compare favorably with the compendium of 124 TF motif predictions presented in MacIsaac et al. [29]: of the 77 TFs with predictions in each set, 59 (77%) are similar (see Methods). In addition, we predict a different motif for 25 of our significant TF–condition motifs, representing 22 TFs (Figure S5). It should be noted that the number of similar and different motifs combine to be more than the total number of TFs with predictions in both sets. This discrepancy is explained by four TFs (MOT3, SFP1, MSN2, and MSN4) for which we predict condition-dependent motifs. These results are summarized in Table 1.

**Table 1:**
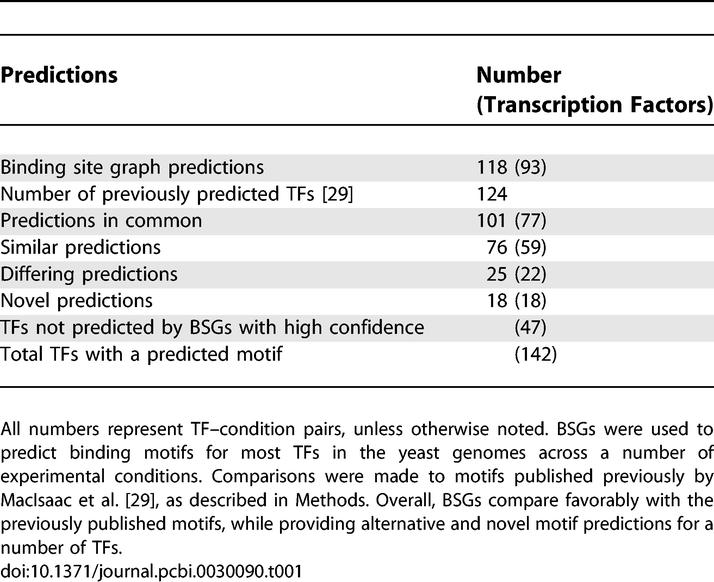
Summary of Genome-Wide Predictions of TF Specificities

Several possible reasons for differences in motif predictions include co-regulation of the same set of genes by different TFs, identification of statistically significant, but biologically inert, motifs, as well as false positive predictions. At the same time, the comparison numbers depend on arbitrary motif similarity thresholds (see Methods). Since allowed degeneracy may be TF-specific, using a single cutoff may not be the optimal approach. However, the agreement provides a rough estimate of the consistency between BSG and previously reported experimental and computational results. Finally, BSGs predict motifs for 18 TFs with previously unknown affinities (Figure S6) and fail to make a significant prediction for 47 TFs. Combining BSG predictions with those in MacIsaac et al. [29] gives a total of 142 TFs with significant motif predictions. However, experimental validation may be needed to confirm novel and revised predictions.

**Figure 5:**
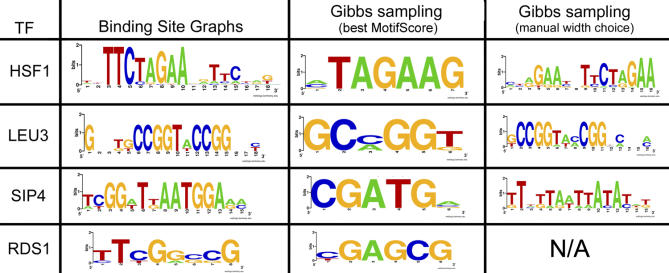
Differences between BSG Predictions and Gibbs Sampling The motif predicted by BSGs is compared with the best-scoring motif from an equivalent amount of Gibbs sampling. In some cases, such as HSF1 and LEU3, BSGs perform better through better estimation of the width of the motif. In such cases, manually choosing the correct motif width based on a priori knowledge allows Gibbs sampling to predict the correct motif. In other cases, however, such as SIP4 and RDS1, choosing the best Gibbs sampling width does not produce the correct prediction. For RDS1, N/A indicates that the motif width reported previously [29] matches the width of the best Gibbs sampling motif, and thus manually selecting the motif width does not alter the Gibbs sampling prediction.

To better understand the reasons behind improved performance of BSGs over traditional Gibbs sampling, we manually examine select significant (*p* < 0.1) BSG predictions that do not agree with the best scoring Gibbs sampling prediction (Figure 5). For Gibbs sampling predictions, we chose the motif width that gave the largest MAP score [17]. Here, we only consider predictions that agree with those previously published. We observed two distinct mechanisms by which BSGs improve on Gibbs sampling. In some cases, such as the HSF1 and LEU3 predictions, the best-scoring result obtained from Gibbs sampling represents only a fraction of the final motif. These represent cases where, through integration of several motif widths, the BSGscore correctly identifies the motif width better than the Gibbs sampling MAP score [17]. Indeed, when we disregard the MAP score and manually choose a motif width according to previous predictions [29], Gibbs sampling identifies the correct motif for HSF1 and LEU3. In other cases, such as SIP4, we find manually choosing the correct motif width does not result in prediction of the correct motif by traditional Gibbs sampling. Similarly, the Gibbs sampling width for the prediction of the RDS1 binding motif matches the width of the previously reported prediction, yet the binding motif does not match. Thus, we conclude that the BSGscore contains additional information about correct binding sites not necessarily present in the MAP score used by Gibbs sampling. In particular, the BSGscore considers both the positional information for each motif and the uniqueness of the nucleotides with respect to the rest of the similarly scoring predictions from the same set of upstream regions. By carefully studying the dynamics of BSG building, it may be possible to incorporate these characteristics into an improved Gibbs sampling procedure and score.

In cases where multiple TFs act together to coordinately regulate a set of genes, numerous motifs may be enriched in a set of promoters. Preliminary evidence suggests these motifs arise as independent connected components in the filtered BSG. For example, BSGs predict two connected components for STE12 in YPD: the first component corresponds to the known STE12 binding motif; the second component is the binding motif for TEC1. STE12 and TEC1 are known to act cooperatively to regulate haploid invasive and diploid pseudohyphal growth. Thus, clustering results into disjoint connected components allowed identification of two different TFs in the same input set. This procedure can be used as a predictor of sets of collaborating TFs in *cis*-regulatory modules. That, in turn, can be used to elucidate major regulatory switches and sets of genes functional in common pathways [30–32].

### Comparison to Other Algorithms

**Figure 6:**
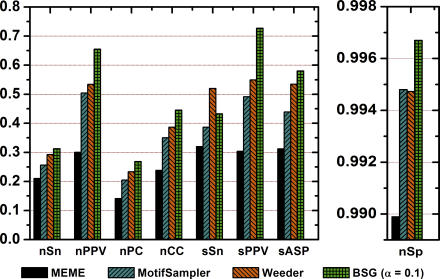
Benchmarked Evaluation of BSG Binding Site Predictions for Yeast Datasets from Tompa et al. [13] Performance of BSG predictions are compared with the three best-performing algorithms according to a previously published evaluation. Performance measures (*x*-axis) are nSn (nucleotide sensitivity), nPPV (nucleotide positive predictive value), nPC (nucleotide performance coefficient), nCC (nucleotide correlation coefficient), sSn (site sensitivity), sPPV (site positive predictive value), and sASP (average site performance). For formulas used to calculate these measures, see Materials and Methods. BSGs significantly outperform all previous evaluated algorithms in nearly every measure. Most notable are improvements in nucleotide and site positive predictive value, where predictions from BSGs achieve values of 0.71 and 0.77, respectively.

We benchmarked performance of BSGs against numerous other motif detection algorithms. We used the same datasets described in Tompa et al. and evaluated BSG predictions according to the statistical framework detailed therein [13]. On yeast-specific benchmark sets, high-confidence (*p* < 0.1) BSG predictions significantly outperform all tested methods according to nearly every statistical measure (Figure 6). In terms of nucleotide correlation coefficient (nCC), an overall measure of correctness, BSG predictions with *p* < 0.1 improve upon the second-best predictions by 19%. The only exception where BSG predictions do not outperform existing techniques is site-level sensitivity (sSn) [11], where Weeder outperforms by 13%. Weeder's sensitivity, however, comes at the expense of many false positive results, as shown by BSGs' significant improvement in sPPV over Weeder (72% versus 55%, a 31% improvement). Moreover, BSG seems to be the only method that predicts many more true positives than false positives, corresponding to a site and nucleotide *PPV* ≫ 0.50. Unlike other methods evaluated, BSG performance does not require any manual curation or custom post processing.

Benchmarking with the mouse and human datasets, we found that the BSG performed among the best six algorithms in every category except nucleotide specificity, for which BSGs performed poorly; while performance was good, we did not observe the broad improvements obtained in yeast (Figure S4). We believe the performance drop in non-yeast sets indicates the need to develop species-specific binding site detection strategies. For example, in the human and mouse tests, the TF binding sites have a positional bias toward the transcription start site; the fly examples, however, tend to contain closely spaced clusters of binding sites. Better understanding of these differences in promoter architecture and usage between species will be critical in developing species-specific BSGscores.

### Robustness to Noise in Input Sequences

Another mechanism to assess the efficacy of a TFBS algorithm is to evaluate the effect of added decoy promoters on the stability and accuracy of TFBS predictions [33]. Decoy promoters are intergenic nucleotide sequences that contain no instances of the TFBS of interest, and may arise through false positives in prediction of the input set. The effect of decoy promoters is reduction in the concentration of TF binding sequences in the input set. For example, 20 instances of a 10-bp binding site in 20 upstream regions, each 1,000 bp long, results in about 1/100 signal:noise. If we add 20 more upstream regions without instances of the same TFBS, signal:noise would be closer to 1/200. Decreasing the signal-to-noise ratio confounds identification of binding sites.

Robustness to decoy sequences is necessary to make binding site predictions from noisy datasets such as high-throughput microarray experiments. Addition of decoy promoters also effectively simulates longer upstream regions encountered in higher eukaryotes. To evaluate BSG robustness to increasing noise, we first predict TFBS in a core set of promoters that ChIP–chip experiments predict are coregulated by a common TF. We then predict binding sites in versions of the core set augmented by increasing numbers of randomly selected intergenic sequences from the S. cerevisiae genome. We then plot PPV with respect to the relative amount of added noise.

**Figure 7:**
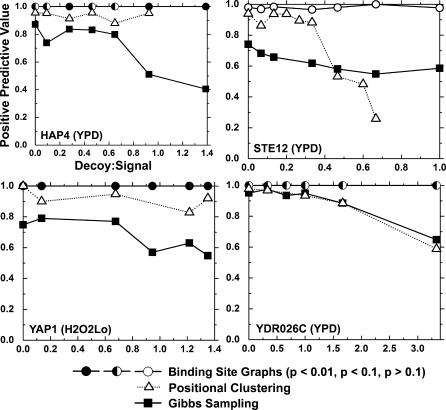
Comparison of Robustness to Noisy Decoy Promoters between BSG Predictions, Positional Clustering [25], and an Equivalent Amount of Gibbs Sampling Runs (6,656 Gibbs Sampling Predictions) For each of the signal sets, varying numbers of random S. cerevisiae promoters were added to the original ChIP–chip derived set (*x*-axis). TFBS predictions were made using BSGs (circles), positional clustering (triangles), and the best predictions from an equivalent number of iterations of Gibbs sampling alone (squares). For each set of predictions, the PPV (*y*-axis) was calculated by comparing the prediction with published motifs as described in Methods. For BSG predictions, filled, half-filled, and open circles represent *p* < 0.01, *p* < 0.1, and *p* > 0.1, respectively. BSGs attain dramatically higher PPV than Gibbs sampling alone, especially in the noisiest input sets. In some cases, the PPV does not decrease monotonically with the addition of noise. This effect is the result of spurious instances of the binding site occurring in the decoy promoters. Although STE12 predictions are not significant, the well-known motif is almost always discovered. In all STE12 predictions, multiple components were identified in the BSG, highlighting the need to generalize the *p*-value to graphs with multiple motifs.

We evaluated robustness of BSG predictions to addition of decoy promoters for four input sets (HAP4, STE12, YDR026C, and YAP1). We then compare our results with those that could be expected given an equal amount of independent Gibbs sampling runs without BSG identification of TFBS. We find, for all TFs evaluated, the PPV of using BSG predictions is uniformly superior to Gibbs sampling alone, even for some predictions with *p* > 0.1. Moreover, the difference between the PPV of BSG and Gibbs sampling alone increases with addition of decoy sequences (Figure 7). We also use the same framework to compare BSGs with positional clustering of frequently recurring Gibbs sampling results [25]. As shown in Figure 7, while positional clustering is useful in improving binding predictions using ensemble Gibbs sampling, BSGs allow further improvement. These results indicate BSGs perform better with noisy input sets that could result from long eukaryotic upstream regions or inaccurate predictions of co-regulation.

### Specificity Determining Positions for E-Box TFs

We found that BSG predictions for the PHO4, CBF1, and TYE7 TFs are particularly interesting. Despite regulating biologically different processes, all three are Helix–loop–helix proteins that bind the hexameric E-box motif CACGTG. In the case of PHO4 and CBF1, a high-throughput microfluidics platform able to precisely measure low-affinity TF:DNA interactions [34] revealed differences in the specificity for E-box flanking nucleotides for PHO4 and CBF1. Previous computational studies [5,29], however, have struggled to identify significant differences in binding affinities. In agreement with the experimentally derived specificities, the BSG is the first high-throughput computational approach able to correctly resolve the differences in specificity of flanking nucleotides for both PHO4 and CBF1 (gCACGTGG and gTCACGTG, respectively, Table S1). Additionally, BSGs predict an extended TYE7 binding 10mer (cATCACGTGa, Table S1) that differs from both the PHO4 and CBF1 binding motifs in the flanking nucleotides. We searched all yeast promoters for exact matches to the expanded binding motifs. As expected, promoters containing the PHO4 motif were significantly enriched in phosphate transport processes. Exact matches to the revised CBF1 and TYE7 motifs were both significantly enriched in amino acid metabolism; CBF1 motifs, however, were limited to metabolism of nitrogen R-groups, whereas TYE7 motifs were limited to metabolism of cysteine. We take this as preliminary evidence that the newly discovered flanking nucleotides may play a major role in allowing each E-box binding TF to regulate a subset of functionally specific proteins.

## Discussion

Here, we present a novel approach for determining the positions and binding affinities to TFs using putatively bound upstream promoter sequences. BSGs are a departure from traditional sequence alignment techniques such as Gibbs sampling primarily because BSGs capture global properties of promoter input sets that seem to be unique only to sets that share TFBS. This results in several important advantages in predicting TFBS using BSGs. First, according to most independent validation criteria, BSGs are more accurate than existing techniques. Additionally, we find BSGs are more robust to noisy decoy sequences than Gibbs sampling with and without positional clustering. Importantly, positional clustering provides an intermediate level of improvement over Gibbs sampling alone. This result suggests the improved performance of BSG is due to a combination of ensemble sampling and analysis of graph-theoretical properties of BSGs [25]. Robustness to decoy sequences may allow BSGs to better predict binding sites from co-expression data, which is more prone to false positive predictions than ChIP–chip, and does not necessarily result in gene sets co-regulated by a single TF. Second, BSG construction and cluster extraction algorithms provide an unbiased estimation of motif width that is better than those based on currently available scoring functions. This can be seen from examples with HSF1 and LEU3 (Figure 5). Finally, comparing BSGscores to a background distribution from graphs constructed for random sets of promoters enables calculations of statistical significance and identification of promoter sets lacking significant motif enrichment or alternatively those that have a high level of noise.

In agreement with earlier observations in synthetic data [35], our results suggest that unlike random promoter sequence sets, input promoter sets enriched in binding by a common TF have densely connected clusters in sequence space. While we chose to use a simple formulation of the weighted clustering coefficient to identify these clusters, other graph clustering approaches can be used to improve binding specificity predictions from BSGs. In their previous work, Pevzner and coworkers suggested using graphical models to predict TFBS [35]. In that work, the authors dissected a simple formulation limited to exactly one binding site per promoter, a fixed-motif width, and a maximum number of mutations per binding site. The authors proposed using graphs that form cliques to identify TFBS. While useful formulations from a theoretical perspective, constraints presented in that paper are limiting from a practical point of view. Our BSG approach does not make any of the above assumptions on motif structure or occurrence. Thus, we were able to apply BSGs to real datasets and successfully identify binding positions with superior accuracy.

The graph-construction technique described here uses ensemble Gibbs sampling across a range of motif widths. We observed that sampling at widths close to the biologically relevant motif width will contribute higher-edge weights to the final graph than sampling far from the biological motif width, which mostly contributes nucleotides at the edges. Combining predictions for each motif width, we can predict the width of the biological motif. According to case studies of LEU3 and HSF1, this strategy results in more accurate identification of motif widths as compared with existing scores. Alternatively, we could evaluate a graph for each possible motif width, and select predictions from the best-scoring graph. Constructing a graph for each motif width, however, would require the ensemble sampling procedure to be repeated many times (once for each width of interest). Doing so is computationally infeasible with available technology; we leave a comprehensive analysis of this strategy for a future study.

Ultimately, using ensemble Gibbs sampling to build BSGs is limited by the sensitivity of Gibbs sampling; thus, constructing BSGs using sampling from more sensitive, faster, or a combination of algorithms [25] may improve performance. BSGs can also aid in integrating ensemble sampling with diverse biological data such as distance from transcription start site; histone localization; free radical cleavage; DNA bending; and phylogenetic conservation into a coherent, unified framework for identifying TFBS [36].

Finally, we used BSGs to predict nucleotide specificity for the majority of TFs in the S. cerevisiae genome using input sets generated from the recently performed whole-genome ChIP–chip experiments. We found some interesting patterns that may be used to control the quality of the data or further our understanding of the interactions between coordinately acting TFs. For example, numerous sets of TFs, based both on our predictions and those of other independent studies, have very similar nucleotide specificity (for example: STE12 and DIG1; PHO4, CBF1, and TYE7). In the case of STE12 and DIG1, protein domain analysis indicates the lack of a known DNA binding domain in one of the proteins (DIG1), and experimental evidence shows that STE12 and DIG1 physically interact [37]. As such, it is likely that DIG1 does not directly bind DNA, but instead co-precipitates with STE12 through formaldehyde crosslinking of protein–protein interactions in ChIP–chip experiments [38,39]. Motif similarity may also stem from cooperative or competitive binding between the factors. Importantly, the increased accuracy of BSG predictions allowed us to predict specificity-determining nucleotides for the E-box TFs PHO4, CBF1, and TYE7 [34]. Two out of the three extended predictions were independently confirmed by recently published experimental results. The third is awaiting further validation.

## Materials and Methods

### Ensemble Gibbs sampling.

We use a threshold Gibbs sampling strategy similar to BioProspector [17]. Briefly, the threshold sampling strategy uses a high threshold to allow inclusion of multiple sequences per promoter, and a low threshold to allow reporting of no sequences in a promoter. The high threshold is set proportional to the product of the average promoter length and the window width, while the low threshold is initialized to 0, and increased linearly to an upper bound. For a complete description of threshold sampling, see Liu et al. [17]. Additionally, we include a third-order background model from genomic promoters, and a modified motif score (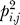 instead of *p_i,j_*), which we found better emphasized conservation within motif predictions:

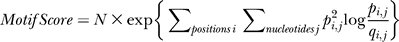
where *N* is the number of aligned segments in the motif, *p_i,j_* is the probability of nucleotide *j* at position *i* in the motif, and *q_j_* is the probability of nucleotide *j* in the third-order background [17]. We sample until stability (predictions do not change over 1,250 updates) 60 iterations and select the single best-scoring motif observed as a single prediction. We found running the sampler for more stable updates did not significantly alter results.

To evaluate the ensemble Gibbs sampling predictions for a set of input promoters, we mask low-complexity sequences, and proceed to collect 512 Gibbs sampling predictions at each motif width from 6–18 bp. In total, 6,656 binding site predictions are collected from 60 × 6656 = 399,360 Gibbs sampling iterations. The number of predictions used was selected to ensure stability in graph construction, and we found performance deteriorated significantly when constructing graphs from fewer sampling predictions. High-performance computing was utilized to perform the ensemble sampling, requiring between 30 min and 5 h of running time on 1024 × 700 Mhz PowerPC 440 processors. While BSG construction currently requires access to high-performance computing, advancements in the algorithm, Gibbs sampling, and computer technology may all help to make the approach more accessible.

### Binding site graph.

A BSG is a weighted, undirected graph *G*: = (*V*,*E*) where each vertex *v* ∈ *V* corresponds to a nucleotide in the input set of promoters, and each edge *e* ∈ *E* indicates the alignment of a pair of nucleotides in a binding site for the same TF. Each edge *e* has weight *w_e_* that measures the similarity between nucleotides as estimated using Gibbs sampling.

We also introduce a threshold BSG, constructed by removing all edges with weight less than threshold *ρ* ∈ [0,1] from graph *G*. Formally, *G_ρ_*: = (*V_ρ_*,*E_ρ_*) where *E_ρ_* ⊆ *E* and *e* ∈ *E_ρ_* ↔ *w_e_* ≥ *ρ*; and *V_ρ_* ⊆ *V* and *v* ∈ *V_ρ_* ↔ {*v* has at least one edge in *E_ρ_*}.

### Binding site graph construction.

For a set of input promoters, we initialize the BSG with one vertex for each nucleotide in the input set, and with no edges (Figure 1A). We evaluate the ensemble behavior of Gibbs sampling over the input. For each pair of aligned nucleotides in each sampling result, we add a unit weight edge between the corresponding vertices in the BSG (Figure 1B). Therefore, if a binding site prediction aligns *N* segments, each *w* nucleotides long, we add *w* · *n*(*n* − 1) / 2 edges to the BSG. If an edge already exists, we instead increase the edge weight by 1. Thus, we weigh each edge by the number of times ensemble Gibbs sampling predictions align the corresponding nucleotides (Figure 1C). After collecting edges from all predictions, we normalize edge weights to [0,1] through division by the maximal possible edge weight (i.e., the number of Gibbs sampling results collected).

### Weighted clustering coefficient.

For a vertex *k*, let *v* be the number of vertices adjacent to *k*, and let *t* be the number of triangles containing *k*. The clustering coefficient [28], is defined as:

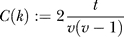



Intuitively, the clustering coefficient is the probability that any two vertices adjacent to *k* have an edge between them. In the dense extreme, when *k* resides in a clique, all vertices adjacent to *k* have an edge between them and the clustering coefficient is 1. In the sparse extreme, when *k* resides in a tree, no edge exists between any two neighbors of *k* and the clustering coefficient is 0. The clustering coefficient is undefined when *k* has less than two adjacent vertices; in such cases, we let *C*(*k*) = 0.

Numerous generalizations of the clustering coefficient to weighted graphs have been proposed [40,41]. We use a definition that weights each triangle by its intensity [27]:

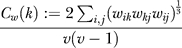
where *w_ij_* is the weight of the edge connecting vertices *i* and *j*. The weighted clustering coefficient of a graph *G* is the average weighted clustering coefficient over the vertices in *G* [28]:

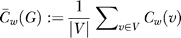



### Binding site graph TFBS prediction.

We predict binding sites from a BSG *G* using a two-step process. First, we select a threshold ρˆ that maximizes the BSGscore,

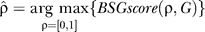
where 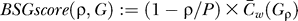 , P is the maximal edge weighted observed in *G*, and 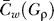 is the mean weighted clustering coefficient of the BSG filtered at *ρ* (see above). A final BSG 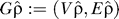 is then created by discarding all edges with weight < ρˆ, and the remaining nucleotides (*V* ρˆ) are collected. To convert the collected nucleotides into binding sites, nucleotides adjacent in the original input promoters are joined together into contiguous segments. The segments then serve as seeds for TF binding site predictions. We dust-filter single nucleotide segments from the seeds, and expand the remaining seeds according to a seed extension threshold *τ*. To do so, we evaluate the strength of each nucleotide *n* in the unfiltered BSG,

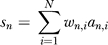
where *w_n_*_,*i*_ is the edge weight between nucleotides *n* and *i*, and *a_n_*_,*i*_ is a delta function equal to 1 when an edge exists between *n* and *i*, and equal to 0 otherwise [41]. The strength of each nucleotide is normalized to *s_i_* ∈ [0,1].

Nucleotides adjacent in the original input promoters are grouped together into seed sequences. Initial seeds are then extended to include adjacent 5′ and 3′ nucleotides with s > *τ*. At the most sensitive extreme (*τ* = 0), initial seeds are maximally extended by including all neighboring nucleotides identified by Gibbs sampling. At the most specific extreme (*τ* = 1) the initial seeds are returned, without extension, as predictions. Between these extremes, a tradeoff between sensitivity and PPV is made (Figure S2). For the purposes of this study, we used a seed extension threshold of *τ* = 0.7, but other values may be more appropriate for different research needs. Lastly, the extended seeds are dust-filtered to remove predictions 6 bp or shorter.

### Performance evaluation.

We evaluate BSG performance using the datasets and statistical measures described in Tompa et al. [13]. Briefly, Tompa et al. create a number of synthetic and real input promoter sets with known binding sites. While the study evaluates input sets from four species (Homo sapiens, Mus musculus, Drosophila melanogaster, and S. cerevisiae), performance of each tool on non-yeast datasets was dramatically worse and for poorly understood reasons. Thus we limit our evaluation to the better understood S. cerevisiae input sets. We predict binding sites for each input set, and evaluate results according to a number of statistical measures. We compare performance in each measure with the published evaluations of 13 existing methods.

We calculate statistical measures as follows. At the nucleotide level, true and false positives and negatives (nTP,nTN,nFP,nFN) are counted through comparison with nucleotides in the known binding sites in each input set. At the site level, true positives, false positives, and false negatives (sTP, sFP, sFN) are counted. A true positive (sTP) is defined as a predicted site that overlaps a known site for at least 25% of the known site.

Based on these counts, we calculate the following nucleotide (*x* = *n*) and/or site (*x* = *s*) level measures:

Sensitivity: *xSN*: = *xTP*/{*xTP* + *xFN*}

Positive Predictive Value: *xPPV*: = *xTP*/{*xTP* + *xFP*}

Specificity: *nSP*: = *nTN*/{*nTN* + *nFP*}

Correlation coefficient [42]:

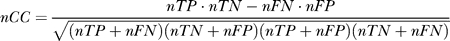



Performance coefficient [35]: *nPC*: = *nTP*/{*nTP* + *nFN* + *nFP*}

Average Site Performance: *sASP*: = {*sSn* + *sPPV*}/2

For a more detailed discussion of statistical measures used, see [13].

### Motif–motif alignment.

We use a PWM to represent TF binding motif predictions [43,44]. To align PWMs, we use a dynamic programming implementation of a modified ungapped local sequence alignment [45] similar to that of Pietrokovski [46]. Similarity between positions in two motifs was measured using Pearson's correlation coefficient:

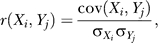
Where *X_i_* is the distribution of nucleotides at position *i* in motif *X*; σ_*X*_*i*__ is the variance at position *i* in motif *X*; and cov(*X_i_*,*Y_j_*) is the covariance of nucleotides at position *i* in motif *X* with nucleotides at position *j* in motif *Y*. Alignment scores range from 0, representing no positions aligned, to the length of the shorter of the two motifs, representing a perfect match between the two PWMs.

A BSG PWM was considered to match to a previously published PWM if the ratio of the above alignment score (i.e., the optimal local Pearson's correlation coefficient) to the information content of the previously published PWM [47] is greater than 0.375. It is important to note that the correlation coefficient is at most *w*, with width of the alignment, whereas the mutual information is at most 2*w*.

### Robustness to noisy sequences.

We use ChIP–chip assays [38] to identify sets of S*. cerevisiae* promoters bound with high confidence (*p* < 0.001) by the TFs STE12, HAP4, and YDR026C in YPD growth media; and YAP1 in low hydrogen-peroxide conditions [5]. For each set, we collect promoter sequences (up to 1 kb upstream) to serve as a seed input for binding site predictions.

We construct noisy input sets by supplementing each seed set with increasing numbers of randomly chosen S. cerevisiae promoters. We predict TF binding sites in the seed and noisy sets using both BSGs and Gibbs sampling. We label results as true or false positive (TP,FP) according to motif–motif alignment scores between the predicted and the known TF binding motif [25], and calculate the PPV:

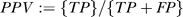
i.e., the percentage of predictions similar to the known binding motif.

### Genomic S. cerevisiae TF binding motif prediction.

We use ChIP–chip data [5,6] to create input sets for all TFs under every condition studied, as described previously. We use BSGs to predict TF binding motifs for each set containing more than four bound probes. The results of the genomic study are available online at http://cagt10.bu.edu/BSG.

## Supporting Information

Figure S1 ROC Curve of Binding Site Predictions Made by Selecting Nucleotides Connected by Highly Weighted Edges BSGs were constructed for all yeast input sets from Tompa et al. For each data point, an edge weight threshold was selected, and all edges with lower weight were removed from the BSGs. Isolated nucleotides were discarded, and the remaining nucleotides used as TFBS predictions. Sensitivity and specificity were evaluated as described in Tompa et al. (see Methods), and averaged over all BSGs. Increasing the edge weight threshold results in nearly uniform increase in PPV (inset), and a corresponding improvement in specificity at the expense of decreased sensitivity.
(267 KB TIF)

Figure S2 Effect of Seed Extension Threshold on Nucleotide Sensitivity and on Nucleotide Positive Predictive Value BSGs at varying seed extension thresholds (*x*-axis) were used to predict TFBS in a series of real and synthetic datasets constructed by Tompa et al. [13]. Increasing the seed extension threshold exchanges nucleotide sensitivity (open triangles) for nucleotide PPV (closed triangles). Variations in the threshold had little or no effect on site sensitivity or site PPV (unpublished data).
(218 KB TIF)

Figure S3 Behavior of the Mean Weighted Clustering Coefficient in BSGs for Signal Sets (Colored Lines with Symbols) and Control Sets (Black Lines) At each edge weight threshold (*x*-axis), edges with subthreshold weight are removed from the graph, and isolated nucleotides discarded. The weighted clustering coefficient is averaged over the remaining nucleotides (*y*-axis). Two trends are evident. First, signal sets uniformly attain a higher maximal weighed clustering coefficient (inset). Second, there is generally a positive correlation between mean clustering coefficient and edge weight thresholds in signal sets.
(106 KB TIF)

Figure S4 Receiver Operating Characteristic of BSG Performance in Yeast, Mouse, and Human Benchmarks Points were collected at each level of statistical significance. While BSGs are predictive for each organism, performance on Yeast promoter sets is superior to that on mammalian systems.
(115 KB TIF)

Figure S5 BSG Prediction of 11 Significant (*p* < 0.1) Motifs That Differ from Previously Published Predictions [29] Differences in prediction may be due to erroneous prediction, coordinated regulation by multiple (possibly physically interacting) TFs, or condition-dependent TF specificities.
(645 KB TIF)

Figure S6 Novel TF Binding Specificities Predicted Using the BSG Framework Detailed Herein In total, BSGs predicted 53 TF specificities for which no previous predictions exits. Displayed are 17 such predictions with *p* < 0.1.
(645 KB TIF)

Table S1 Resolving the Nucleotide Specificity of E-Box Binding TFs The yeast TFs PHO4, CBF1, and TYE7 are all known to bind E-box motifs but regulate distinct functions. For each factor, the BSG predicts distinct nucleotides flanking the core E-box hexamer CACGTG that may be useful to confer E-box motif specificity to these TFs.
(27 KB DOC)

## Abbreviations

BSG: binding site graph
BSGscore: binding site graph score
PPV: positive predictive value
PWM: position weight matrix; transcription factor
TFBS: transcription factor binding sites
