# Supplementary material for: Binding Site Graphs: A New Graph Theoretical Framework for Prediction of Transcription Factor Binding Sites
Source: PLoS Comput Biol. 2007 May 11;3(5):e90. doi: 10.1371/journal.pcbi.0030090 (PMC1866359; doi:10.1371/journal.pcbi.0030090)
Supplement: Supplementary file 1 [file pcbi.0030090.st001.doc]

# Table S1:

|  | PHO4 | CBF1 | TYE7 |
| --- | --- | --- | --- |
| Binding Site Graphs | gCACGTGG | gTCACGTG | cATCACGTGa |
| MacIsaac et al, 2006 | CACGTG | GTCACGTG | tCACGTG |
| Harbison et al, 2004 | CACGTG | CACGTG | tCACGTGa |
